# Exploring the potential impact of group identity on post-traumatic growth in the aftermath of Corona outbreak: function of social–emotional competence as a mediator

**DOI:** 10.3389/fpubh.2023.1282462

**Published:** 2023-10-12

**Authors:** Jinfu Ma, Ahsan Riaz Khan, Hai-Jun Zhang, Zhang Jun, Mohamed R. Abonazel, Muhammad Salman Ahmad, Elsayed M. Tageldin, Ali Rashash R. Alzahran

**Affiliations:** ^1^Pakistan Studies Center, North Minzu University, Yinchuan, China; ^2^Department of Interventional and Vascular Surgery, Shanghai Tenth People's Hospital, Tongji University School of Medicine, Shanghai, China; ^3^National United Engineering Laboratory for Biomedical Material Modification, Branden Industrial Park, Qihe Economic and Development Zone, Dezhou, China; ^4^Research Center for Translational Medicine, Shanghai East Hospital, School of Medicine, Tongji University, Shanghai, China; ^5^Shanghai Institute of Stem Cell Research and Clinical Translation, Shanghai, China; ^6^Department of Applied Statistics and Econometrics, Faculty of Graduate Studies for Statistical Research, Cairo University, Giza, Egypt; ^7^School of Culture and Tourism, Qujing Normal University, Qujing, China; ^8^Electrical Engineering Department, Faculty of Engineering and Technology, Future University in Egypt, New Cairo, Egypt; ^9^Department of Mathematical Sciences, College of Applied Sciences, Umm Al-Qura University, Mecca, Saudi Arabia

**Keywords:** COVID-19, social–emotional competence, mental wellbeing, human-society relationship, post-traumatic growth

## Abstract

**Background:**

This research endeavors to examine the potential effects of human and societal interactions on individuals’ post-traumatic growth in the aftermath of the Corona outbreak. To achieve the aforementioned objective, the current research investigates the correlations between post-traumatic growth and group identity, while also examining the potential mediating function of social–emotional competence.

**Methods:**

A cross-sectional design included a representative sample of 2,637 high school students located in the capital territory of Pakistan using convenience sampling method. To explore the associations, correlation and mediation analyzes utilizing the group identification scale, the social–emotional competence scale, and the post-traumatic growth scale was performed with SPSS PROCESS 4 macro and AMOS.

**Results:**

The findings demonstrated that group identification emerged as a substantial predictor substantially associated with post-traumatic growth. Moreover, the relationship linking group identification and post-traumatic growth was found to be partially moderated by social–emotional competence.

**Conclusion:**

The phenomenon of group identification can exert influence on post-traumatic growth through both direct and mediating pathways, with the latter being essentially mediated by social–emotional competence. The aforementioned outcomes possess significant academic and practical implications concerning the promotion of post-traumatic growth and the improvement of psychological well-being after the Corona outbreak.

## Introduction

There have been multiple scholarly investigations indicating that the ongoing Corona outbreak has posed significant adverse consequences on individuals’ psychological well-being, eliciting various psychological symptoms such as anxiety, depression, and post-traumatic stress disorder (PTSD) (1, 2). This impact has been especially pronounced among adolescents and college students, who have faced a range of challenges including but not limited to heightened levels of depression, anger, and sleep-related issues (3). Considering the potential enduring psychological influence of the pandemic, it is of utmost importance to investigate strategies aimed at assisting the general populace in effectively managing the psychological ramifications of Corona outbreak, while also fostering post-traumatic growth in the era following the pandemic. Post-traumatic growth elucidates the salient psychological transformations that may transpire within an individual when confronted with adversity or comparable challenges (4). Notably, these transformative changes encompass an enhanced sense of gratitude toward life, heightened levels of personal fortitude and self-perception, augmented interpersonal connections, and more profound personal encounters (5). A comprehensive examination of existing scholarly works has revealed that post-traumatic growth is closely tied to individuals’ psychological emotions, rumination, coping mechanisms, and behaviors (6). Furthermore, numerous research investigations have consistently demonstrated that PTG experienced amidst the corona outbreak is significantly connected to individuals’ expectations of life, sense of societal and collective ties, and overall mental well-being (7, 8).

Several aspects exert a substantial impact on an individual’s post-traumatic growth, encompassing both intrinsic aspects as well as extrinsic aspects (9–12). Furthermore, it is widely acknowledged that group identity has a pivotal part in influencing people’s post-traumatic growth (13). Group identity is a psychological notion that signifies the identification and emotional ties people develop toward a specific community ((14)). This attachment and identification are contingent upon the presence of common traits or circumstances. Ultimately, this process fosters a greater feeling of being related within the community. Limited empirical research has thus far delved into the correlation between group identity and post-traumatic growth. Nevertheless, extant scholarship has demonstrated a robust connection between these two constructs. An empirical investigation indicated that conventional cognitive frameworks acted as obstacles in establishing an effective link with the collective, thereby culminating in adverse affective states that obstructed post-traumatic growth (15). Henson and colleagues discovered that the presence of attachment avoidance within a certain group of academic instructors and students impeded their ability to experience post-traumatic growth (16).

There has been a noticeable increase in scholarly attention toward the significance of social–emotional competence in the regulation of behavioral and cognitive processing (17). Social–emotional competence is conceptualized as an individual’s capacity to cognitively recognize and effectively communicate emotions, while also possessing the capacity to comprehend and adeptly utilize emotions across a diverse range of circumstances. Additionally, there exists a robust association between the perception of emotion, logical thinking, and the regulation of emotion (18). Social–emotional competence serves as a crucial contributing factor in facilitating the process of post-traumatic growth. In the realm of scholarly research, investigations were conducted to analyze the emotional competence exhibited by adolescents affected by the global pandemic, coronavirus epidemic, both while in isolation and after its conclusion (19). The findings of these studies substantiate the assertion that heightened social–emotional competence can contribute positively to the process of post-traumatic growth among adolescents. Moreover, multiple studies have demonstrated a positive correlation between self-esteem and general intelligence (20, 21). An illustration of this phenomenon can be observed through research findings revealing that a robust gastrointestinal system is correlated with an elevated state of contentment, subsequently augmenting individual self-esteem and confidence. The literature lacks sufficient exploration of the mediating function of social–emotional competence in the connection between these factors, despite their inherent interconnectedness. Hence, following the advent of the coronavirus outbreak, it becomes imperative to explore how human-society interactions can support individuals in managing the psychological distress caused by Corona disease and foster the development of post-traumatic growth.

To achieve this objective, a comprehensive survey was administered to ascertain the perspectives of secondary education students who have been affected by the Corona outbreak. The primary goal of this investigation was to explore the interconnections between post-traumatic growth and group identity, while simultaneously examining the potential mediating influence of social–emotional competence. The present study offers significant findings about the mental well-being of adolescents, thus facilitating the development of specific psychological interventions to aid individuals impacted by the pandemic.

By conducting a comprehensive evaluation of the available research regarding group identity and post-traumatic growth, a prospective connection between these two constructs has been recognized. These psychological improvements may then contribute to the development of effective coping strategies for people dealing with traumatic events and stressors. According to research, it has been suggested that fostering community engagement and encouraging people to engage in collective engagements and maintain connections with their societal networks amid the Corona outbreak can serve as a facilitator for the cultivation of prosocial behaviors and augmentation of mental wellness (22). Emerging research has proposed that intervention efforts targeting the relationship between adolescents and their interpersonal connections have the potential to mitigate trauma and foster post-traumatic growth, especially in those individuals impacted by the unprecedented Coronavirus global health crisis. Building upon these findings, our hypothesis posits a positive correlation between post-traumatic growth and group identity, i.e., “*H1*: *Group identity has a positive association with post-traumatic growth”*.

On the contrary, extant scholarship suggests that social–emotional competence confers significant advantages upon individuals, affording them the capacity to effectively acclimate to both the educational environment and societal context. Additionally, social–emotional competence fosters improved educational achievement, the cultivation of essential adaptive competencies, and the promotion of favorable behavioral and cognitive (23, 24). An empirical investigation conducted in Poland examined the impact of corona epidemic on psychological wellness. The findings indicated the presence of a favorable association between individuals’ social–emotional competence and their mental well-being. Specifically, individuals with elevated social–emotional competence were found to exhibit comparatively low degrees of negative emotional states, including anxiety, sadness, and fear (25). A subsequent investigation not only revealed the potential impact of social support and environmental modifications on students’ self-efficacy beliefs but also emphasized their role in moderating such beliefs. The group identity construct presents itself as an additional influential element that has the potential to significantly affect social–emotional competence (26). Research in the literature indicates that social–emotional competence has demonstrable effects on relationships, social interactions, and group identity among adolescents. However, there is widespread consensus that group identity plays a pivotal role in shaping adolescent social–emotional competence (27, 28). The social cognitive notion posits that people engage in the construction of social connections, which subsequently impact their cognitive and interpersonal conduct. Key convictions, including feelings of worth, belongingness, and self-efficacy, are believed to have a significant impact on the evolvement of social–emotional competence. Consequently, the influence of group identity on these perceptions may either facilitate or impede the process of constructing individual social–emotional competence. In light of the aforementioned observations, it is hypothesized that a direct and favorable association exists between group identity and social–emotional competence, i.e., *“H2: group identity has a positive association with social–emotional competence”*.

The Corona outbreak has engendered a growing amount of scholarly research that explores the correlation between social–emotional competence and psychological wellness (29). A research study determined that children possessing elevated levels of social–emotional competence demonstrated less occurrence of cognitive and behavioral difficulties (30, 31). Correspondingly, additional evidence emerged affirming that heightened social–emotional competence aids individuals in enhancing their self-efficacy, job satisfaction, and psychological wellness. According to the emotional intelligence theory, people’s level of intellectuality can potentially be manifested through their proficiency in four crucial domains, specifically the discernment of emotions, comprehension of emotions, articulation of emotions, and management of emotions. This paper aims to elucidate the moderating impact of social–emotional competence on human psychology (18). Based on a thorough examination of the existing scholarly literature, it is our assertion that a growth mindset significantly impacts post-traumatic growth. Furthermore, we have discovered a noteworthy significant connection between post-traumatic growth, group identity, and social–emotional competence, and. Based on the aforementioned foundation, we put forward the proposition that: *“H3: Social–emotional competence has a mediation effect on post-traumatic growth and growth identity”*.

## Materials and methods

### Participants and procedures

This research focused on a cohort of freshmen, specifically those who had been affected by the coronavirus outbreak. The students were selected from the capital territory of Pakistan and fell within the age range of 16 to 18. A sample comprising 2,987 students was chosen as the subjects for the study using the convenience sampling method. Questionnaires in paper format were dispensed to the designated respondents during the period spanning from March to June 2022, after the Corona outbreak. Before commencing with the administration of the questionnaire, the researcher elucidated the purpose and specifics of the research and survey questions to the students partaking in the research. The questionnaires were disseminated after obtaining consent from the parents, classroom teachers, and students, who were subsequently given instructions to complete them. Subsequently, the questionnaires were duly gathered and the acquired data was subsequently inputted. Following the conclusion of data collection, the researcher proceeded to assess the questionnaire’s validity. Subsequently, 2,637 questionnaires were deemed valid, after eliminating those with irregular or missing responses. This resulted in a commendable recovery rate of 88.28%.

## Materials

The questionnaire used in this investigation consisted of four distinct categories, namely: a social-demographic information tool, a scale measuring group identity, a scale assessing post-traumatic growth, and a scale gaging self-efficacy and coping. The demographic information questionnaire solicited data regarding the respondents’ sex, place of residence (PoR), and SES.

### Group identity scale

The present investigation utilized the Group Differences in Industrial Organizations (GDIO) scale constructed by Brown et al. (32) to evaluate the extent of group identification. In order to guarantee excellence and reliability, the conclusive questionnaire was comprised of a set of five items. In this metric, individuals are presented with a series of five inquiries designed to evaluate the intensity of their collective affiliation with their peer associations and educational cohorts. Every question on the survey received a positive score on a 5-point Likert scale, except one, which received a negative score. The internal consistency reliability of the measure, as measured by Cronbach’s alpha coefficient, was moderately high at 0.89 (Table 1).

**Table 1:** Reliability analysis.

Constructs	Cronbach’s α
Group identity	0.89
Post-traumatic growth	0.82
Social emotional competence	0.88

### Social–emotional competence scale

This scale has been widely used to assess social–emotional competence and consists of 12 items, divided into four distinct categories (33). The questionnaire is rated on a 7-point Likert scale with higher values exhibiting intense levels of pro-social behavior among students. The internal consistency reliability of the measure, as measured by Cronbach’s alpha coefficient, was moderately high at 0.88 (Table 1).

### Post-traumatic growth scale

This indicator was derived from the initial scale devised by Cann et al. (34) The adaptation of the scale was necessitated by the variances in cultural contexts and the cognitive maturation of adolescent populations. The questionnaire in its ultimate iteration consisted of four distinct dimensions. The survey consisted of a total of eight inquiries, wherein the respondents’ responses were evaluated using a 6-point Likert scale. Higher scores on this scale were indicative of higher levels of post-traumatic growth reported by the participants. The internal consistency reliability of the measure, as measured by Cronbach’s alpha coefficient, was moderately high at 0.82 (Table 1).

### Covariates

In accordance with existing empirical investigations highlighting the potential correlation between an individual’s gender, PoR, and social–emotional competence with post-traumatic growth, we incorporated these factors as covariates within our research framework.

### Analytical approach

Data analysis for the present investigation was done using AMOS and the SPSS (v25). Harman’s single-factor test procedure was initially employed to assess for common method bias to ensure the quality of the analyzes (35) (36). Pearson correlation analysis and descriptive statistics were also assessed for the selected study participants to ensure normality and no multicollinearity in the data. Then, AMOS was utilized to develop the structural equation model (SEM) and ensure the accuracy of the measurement model. The research hypotheses were tested using the SPSS PROCESS 4 macro. Furthermore, we conducted a rigorous assessment of the hypothesized model using the bootstrapping technique (involving *N* = 10,000). Group identification was considered as the explanatory variable, while post-traumatic growth served as the response variable. Social–emotional competence was identified as the mediator, and sex, PoR, and SES were accounted for as covariates.

## Results

The survey-based approach could potentially result in common method bias, hence before conducting data analysis, Harman’s one-way test was used to examine all items in the questionnaire. The findings indicated the presence of 12 factors with eigenvalues surpassing the value of one. The percentage of maximum factor variance explained was only 19.34%, which is significantly lower than the standard threshold. This suggests that there was no notable common method bias present.

Table 2 shows the basic descriptive attributes of the study participants whereas Table 3 illustrates the Pearson’s correlations among the participants. Among the 2,637 participants included in this study, a majority of 1747 individuals (66.25%) identified as females, while 890 individuals (33.75%) identified as males. Regarding the individuals’ PoR, 1789 individuals (constituting 67.84%) were situated in urban areas, while 848 individuals (accounting for 32.16%) resided in rural locations. Further, we examined the potential connections between participants’ sex, PoR, and SES with social–emotional competence and post-traumatic growth by assessing the connections between these factors and the key factors. The findings indicated that only sex and PoR had a connection with SES, whereas SES was significantly linked to group identity, social–emotional competence, and post-traumatic growth. In the meantime, there was a substantial and positive correlation between post-traumatic growth, group identity, and social–emotional competence, and. In addition, the results of Pearson correlation coefficients confirmed the absence of multicollinearity among study variables as no coefficient exceeded 0.7.

**Table 2:** Descriptive analysis.

			$\bar{X}\pm SD$	Significance	Effect size
Group identity			21.68 ± 4.95		
	Sex			0.04	0.10
		Male	21.42 ± 5.11		
		Female	22.39 ± 4.23		
	*PoR*			<0.01	0.25
		Urban	24.14 ± 4.84		
		Rural	24.05 ± 4.26		
Post-traumatic growth			77.11 ± 12.45		0.40
	*Sex*			<0.01	
		Male	76.85 ± 14.05		
		Female	75.43 ± 12.05		
	*PoR*			<0.01	
		Urban	77.45 ± 11.24		
		Rural	71.18 ± 9.51		
Social–emotional competence			61.52 ± 9.53		0.40
	*Sex*			0.03	
		Male	60.44 ± 9.74		
		Female	61.85 ± 8.88		
	*PoR*			0.02	
		Urban	61.72 ± 9.15		
		Rural	60.21 ± 9.76		

PoR, place of residence, $\bar{X}$ , mean, SD, standard deviation, significance level *p* < 0.05.

**Table 3:** Pearson’s correlation analysis.

	Sex	PoR	SES	GI	SEC	PTG
Sex	1					
PoR	−0.234*	1				
SES	0.347**	−0.205*	1			
GI	0.149	−0.146	0.217*	1		
SEC	0.258**	−0.284**	0.339	0.469**	1	
PTG	−0.373	−0.147	0.262*	0.437**	0.512**	1

*p < 0.05, **p < 0.01, PTG, post-traumatic growth; SEC, social–emotional competence; GI, group identity; PoR, place of residence; SES, socioeconomic status.

We employed AMOS to conduct a confirmatory factor analysis and assess the validity of our primary constructs. The simple mediator model that was proposed resulted in appropriate model adequacy. The confirmatory factor analysis study indicated that the measurement model was suitable, with acceptable fit indices: RMSEA = 0.031, SRMR = 0.030, CFI = 0.96, and TLI = 0.97. In the context of analyzing a simple mediation model with explicit variables, the utilization of SPSS PROCESS proves to be an uncomplicated and efficient approach (Table 4). It is considered more appropriate for conducting mediation analyzes with variables of an explicit nature as compared to AMOS.

**Table 4:** Model fit indices.

Indices	Values
$\chi^{2}/df$	1.37 (*p* < 0.001)
RMSEA	0.031
SRMR	0.030
CFI	0.96
TLI	0.97
IFI	0.96

RMSEA, root mean square error of approximation; SRMR, standardized root means square residual; CFI, comparative fit index; TLI, Tucker-Lewis’s index; IFI, incremental fit index.

Furthermore, an analysis employing the SPSS PROCESS tool (model 4) was employed to undertake multiple regression analysis, wherein group identity served as the explanatory variable, post-traumatic growth as the response variable, and social–emotional competence as the mediating factor. As hypothesized, the group identity demonstrated a statistically significant positive predictive influence on post-traumatic growth ( β=.328 , *p* < 0.01) Furthermore, it was observed that there was a substantial and affirmative correlation between group identity and social–emotional competence ( β=.374 , *p* < 0.01). This significant positive correlation further extends to the relationship between social–emotional competence and post-traumatic growth ( β=.458 , *p* < 0.01; Table 5).

**Table 5:** Regression analysis.

	PTG ( $R^{2}$ =0.129)			SEC ( $R^{2}$ =0.348)			PTG ( $R^{2}$ =0.217)		
	*β*	*t*	Sig.	*β*	*t*	Sig.	*β*	*t*	Sig.
GI	0.328	9.26**	<0.01	0.374	12.35**	<0.01	0.219**	8.36	<0.01
SEC							0.458	14.68	<0.01
Sex				0.163	7.25*	0.03	−0.127	−3.58	0.347
PoR				−0.196	−8.729**	<0.01	−0.078	−2.61	0.762
SES				0.203	6.77**	<0.01	0.113	5.65	0.943
*F-statistics*	19.50**			24.49**			29.16**		

*p < 0.05, **p < 0.01, PTG, post-traumatic growth; SEC, social–emotional competence; GI, group identity; PoR, place of residence; β, regression coefficient; SES, socioeconomic status.

Furthermore, the bootstrap method was employed to identify and assess the presence of mediating impacts. After controlling for confounding factors, i.e., sex, PoR, and SES variables, the 95% confidence interval for the mediating effect of group identity on post-traumatic growth through SES and social–emotional competence was observed to be (0.032–0.113). Similarly, the 95% confidence interval for the direct impact of group identity on post-traumatic growth was determined to be (0.101–0.223). Importantly, neither of these intervals contained the value 0, suggesting a statistically significant relationship between these variables (Table 6). It is evident from the outcomes that group identity can have an indirect impact on post-traumatic growth through social–emotional competence. Additionally, group identity also exerts a direct influence on post-traumatic growth, whereby the direct effect constitutes 70.88% of the overall effect, while the mediating effect corresponds to 29.12%. Overall, the research outcomes indicate a significant and positive correlation between the growth mindset of freshmen and their post-traumatic growth. This relationship is mediated by students’ socioeconomic status, thus providing support for the formulated hypotheses.

**Table 6:** Bootstrapping analysis of effect size.

	Effect estimate	Bootstrapped SE	Bootstrapped (95% CI)	Ratio
Direct effect	0.129	0.019	0.101–0.223	70.88%
Mediating effect	0.053	0.011	0.032–0.113	29.12%
Composite effect	0.182	0.021	0.163–0.315	

SE, standard error; CI, confidence interval; significance level at *p* < 0.05.

## Discussion

This study aims to investigate the underlying mechanism between post-traumatic growth and group identity, while also considering the mediating function of social–emotional competence in the aforementioned associations. The population consists of Pakistani adolescents who have encountered the effects of the Corona outbreak. The study outcomes indicate that group identity factors have a constructive impact on post-traumatic growth. Additionally, it was observed that social–emotional competence plays a significant mediating role in the relationship between post-traumatic growth and group identity. The present findings affirm our initial hypotheses and contribute additional insight to the existing body of research by providing a deeper understanding of the correlation between post-traumatic growth and group identity.

The present investigation has elucidated a notable correlation between post-traumatic growth and group identity. This finding aligns with prior empirical investigations that have subsequently substantiated that individuals possessing high levels of group identity tend to also exhibit substantial psychological resilience and optimal mental well-being (37) (38). In our research, we have conducted a comparative analysis of previous studies conducted by different researchers revealing a confluence of findings (10) (39) (40). This confluence provides empirical evidence supporting the proposition that the social identity notion can be utilized as a comprehensive approach for exploring and elucidating the relationship between group identity and post-traumatic growth. The notion addresses this by elucidating the mechanism by which people establish affiliations with societal collectives, elucidating how group identity can function as the foundation for people’s self-esteem, and equipping people with tactics to effectively address and surmount adversities in their personal experiences ((41)). The implementation of these approaches can aid people in effectively managing and coping with adversities (38). Irrespective of a person’s affiliation, enhancing one’s group identity or people’s ability to effectively manage and overcome challenges can prove to be a pivotal determinant. Enhancing one’s sense of belonging and self-worth via elevated group identity or a robust affiliation with any collective can effectively promote both psychological and physical well-being, thereby effectively perpetuating a condition of sound mental health (42) (43). It has been discovered that initiatives targeting the enhancement of group identity have demonstrated a capacity to mitigate the detrimental effects of loneliness stemming from the coronavirus outbreak (44). Moreover, such interventions have exhibited an ability to ameliorate individuals’ sense of belonging and self-worth, while concurrently fostering psychological well-being (45). Moreover, individuals who cultivate a sense of group identity through the utilization of social media platforms amidst the Corona outbreak have conveyed a marked decrease in feelings of isolation and psychological anguish. Furthermore, a strong level of group identity is indicative of lower levels of mental disorders. According to evidence in the field of clinical medicine, it has been found that various factors, such as social support, social inclusion, interventions aimed at improving social relationships, as well as tailored psychological interventions, have the potential to attenuate mental distress and facilitate post-traumatic growth in individuals who have undergone traumatic experiences, such as hospitalization due to Coronavirus (46, 12). These outcomes substantiate the significance of perceived growth and the enhancement of positive mental changes among people.

The results of our investigation add to prior studies on group identity and post-traumatic growth by showing that group identity is a significant determinant of social–emotional competence, supporting earlier conclusions that people who have elevated group identity are likely to have higher social–emotional competence (47, 48). Recent studies have indicated that group identity likely strengthens societal bonding and enhances interactions between individuals (49). For example, Willems and his colleagues discovered that people who have a high level of self-disclosure tend to have strong interconnection, which contributes to their ability to establish and sustain positive connections with others (50). Additionally, a study has revealed that students who form relatively vigorous societal ties tend to gain more social–emotional and cognitive learning through social interaction, resulting in higher levels of social–emotional competence (21, 51). Our research supports the social cognitive notion, which suggests that people form social relationships to shape their emotional and social actions based on their interpretations (52). Thus, perceptions play a significant role in shaping social–emotional competence. According to a study on the Corona outbreak, *Ntontis* discovered that there is a strong connection between group identity, social support, and resilience, with perceived stress showing a negative correlation (53). This suggests that both group identity and social support can influence how individuals perceive things, enabling them to manage their mental well-being, reduce psychological pressure, and ultimately improve their overall socio-economic condition (54, 55). Receiving assistance from one’s social circle including family, friends, and community during stressful situations like the coronavirus outbreak can help individuals feel connected and valued, ultimately enhancing their sense of belonging and self-esteem, while also boosting psychological well-being (56, 57). Hence, strengthening general intelligence is likely vital for enhancing socioemotional competence in individuals.

In addition, our research uncovered a connection that is favorably correlated between SEC and post-traumatic growth. An empirical investigation conducted among adolescents discovered that individuals with high social–emotional competence are more likely to possess a strong ability to cope with challenges and have an optimistic mindset toward difficult situations (58, 59). This reinforces the idea that social–emotional competence plays a significant role in regulating an individual’s mental well-being, as suggested by the emotional intelligence theory. A study showed that people with higher social–emotional competence were better at managing their mental state and emotions during the Crona crisis, allowing them to deal with obstacles constructively (60, 61). Furthermore, people who possess a high socioeconomic status often utilize multiple methods to manage their emotions, cognitive and coping mechanisms (19, 62). They may face challenging circumstances and approach life with positivity by putting constructive strategies into practice. This enables them to discover new options and build a greater knowledge of their value as individuals, ultimately resulting in post-traumatic growth.

In the aftermath of the Corona outbreak, a considerable number of individuals are confronted with substantial psychological distress and trauma. This research establishes a novel connection between group identity, social–emotional competence, and post-traumatic growth, showcasing its significant implications in both theory and practice. This study initially investigates the underlying mechanism between growth from group identity and post-traumatic growth, utilizing the interplay between individuals and the collective as well as individuals and society. This exploration enhances the understanding of post-traumatic growth’s formation mechanism in the aftermath of the Corona outbreak, offering a novel perspective for prospective research endeavors.

Furthermore, our work contributes to the existing body of literature on social–emotional competence and its positive impact on the overall well-being of adolescents. By enhancing their social–emotional competence, adolescents can develop greater comprehension of others’ psychological demands and effectively navigate their own traumatic experiences. These findings also lend support to social identity theory and emotional intelligence theory, thereby reinforcing and expanding their foundational principles. The present work represents a pioneering endeavor in examining the correlation among the above three factors, thereby offering novel insights into comprehending the intricacies and dynamics of posttraumatic growth.

The present study yields several practical significances. To begin with, it is imperative to acknowledge the crucial relationship between group identity and post-traumatic growth. Consequently, it becomes essential to closely monitor the evolution of group identity among adolescents within the wider societal context. Moreover, recognizing the significance of nurturing adolescents’ affiliations with various groups and society at large is paramount, as it aids them in surmounting the lasting repercussions caused by events such as the Corona outbreak or other catastrophic occurrences. As an illustration, the creation of strong societal connections and collective affiliations can aid individuals in alleviating the psychological implications, distress, and tension caused by epidemics and disasters. Furthermore, an effective method to augment the social–emotional competence of adolescents, enabling them to effectively manage traumatic events and distress while fostering their post-traumatic growth, involves the integration of social–emotional education within the curriculum. In the pursuit of fostering individual post-traumatic growth, the incorporation of social–emotional education within the daily curriculum serves as a notable approach. This pedagogical strategy endeavors to encourage students to actively comprehend, articulate, and absorb knowledge, thereby facilitating their ability to navigate personal and social relationships. Notably, this educational initiative assumes a pivotal role in advancing post-traumatic growth after the Corona outbreak. This study offers new insights into the post-traumatic growth process and its mechanisms, consequently enriching and enhancing existing knowledge. Moreover, such findings have practical implications for the field of psychotherapy and counseling, enabling therapists to acquire a comprehensive comprehension of their patients’ requirements and effectively address them.

### Limitations and future directions

The findings of our inquiry necessitate careful consideration of several inherent limitations to appropriately interpret them. The potential introduction of bias in establishing the causality between adolescent group identity, social–emotional competence, and post-traumatic growth could arise due to the cross-sectional nature of the current investigation. In conducting future investigations, it is recommended that researchers employ methodologies such as experimental or longitudinal investigations. These approaches allow for the repeated measurement of adolescents’ behavioral functions and shift over an extended duration. By utilizing these methods, researchers can capture a more thorough and profound understanding as well as draw more conclusive findings. Additionally, it is worth noting that the choice of sample process may exhibit a degree of bias, predominantly stemming from the fact that the questionnaires employed typically target exclusively those adolescents who are accessible within the confines of the capital city of Pakistan. Subsequent investigations can enhance the size of their adolescent samples through the deliberate inclusion of participants sourced from various domains such as family members, educational institutions, communal settings, or diverse geographical regions within Pakistan. This intentional broadening of sample selection aims to mitigate the potential bias inherent in the sampling process. Ultimately, the generalizability of the results may be constrained by the fact that the data originated specifically from a subset of Pakistani high school-aged adolescents. Further research is warranted to ascertain the veracity of these findings among diverse populations, thereby ensuring their applicability and generalizability.

## Conclusion

The objective of this research was to investigate the correlation between group identity and post-traumatic growth after the Corona outbreak, focusing on the interconnections between humans and society. The results suggest that group identity has a direct impact on post-traumatic growth and also indirectly influences it through social–emotional competence. The outcomes of the present investigation offer additional proof of the growth and intensification of the framework and connection between group identity and post-traumatic growth. They also demonstrate how the dynamics of individuals and groups, as well as people and society, impact people’s personal growth and transformation. Smart insights can shape the improvement of public psychological well-being in the era following the Corona outbreak.

## Data availability statement

The original contributions presented in the study are included in the article/supplementary material, further inquiries can be directed to the corresponding authors.

## Ethics statement

The studies involving humans were approved by this study was approved by the Research Ethics Committee of Minzu University (Approval: 2021/0725). The study adhered to the principles of the Declaration of Helsinki. Informed consent was obtained from all the subjects involved in the study. The studies were conducted in accordance with the local legislation and institutional requirements. The participants provided their written informed consent to participate in this study.

## Author contributions

JM: Writing – original draft, Writing – review & editing. AK: Writing – original draft, Writing – review & editing. H-JZ: Funding acquisition, Investigation, Supervision, Validation. ZJ: Data curation, Formal analysis, Supervision, Validation, Visualization. MRA: Data curation, Funding acquisition, Investigation, Methodology, Software, Supervision, Conceptualization, Project administration, Writing – review & editing. MSA: Conceptualization, Data curation, Formal analysis, Supervision, Validation, Writing – review & editing. ET: Data curation, Formal analysis, Investigation, Project administration, Writing – review & editing. AA: Investigation, Software, Supervision, Validation, Writing – review & editing.
